# Insulin-Like Growth Factor Axis Expression in Dental Pulp Cells Derived From Carious Teeth

**DOI:** 10.3389/fbioe.2018.00036

**Published:** 2018-04-12

**Authors:** Hanaa Esa Alkharobi, Hasanain Al-Khafaji, James Beattie, Deirdre Ann Devine, Reem El-Gendy

**Affiliations:** ^1^Division of Oral Biology, Leeds School of Dentistry, St James University Hospital, University of Leeds, Leeds, United Kingdom; ^2^Department of Oral Biology, Faculty of Dentistry, King Abdul Aziz University, Jeddah, Saudi Arabia; ^3^Department of Oral Pathology, Faculty of Dentistry, Suez Canal University, Ismailia, Egypt

**Keywords:** insulin-like growth factor axis, IGF binding protein-3, IGF binding proteins-2, dental pulp stem cells, inflammation, pulp regeneration, caries, dentin

## Abstract

The insulin-like growth factor (IGF) axis plays an important role in dental tissue regeneration and most components of this axis are expressed in human dental pulp cells (DPCs). In our previous study, we analyzed IGF axis gene expression in DPCs and demonstrated a novel role of IGF binding protein (IGFBP)-2 and -3 in coordinating mineralized matrix formation in differentiating DPCs. A more recent study from our laboratory partially characterized dental pulp stem cells from teeth with superficial caries (cDPCs) and showed that their potential to differentiate odontoblasts and/or into osteoblasts is enhanced by exposure to the mild inflammatory conditions characteristic of superficial caries. In the present study, we examine whether changes apparent in IGF axis expression during osteogenic differentiation of healthy DPCs are also apparent in DPCs derived from carious affected teeth.

## Introduction

Dental caries is one of the most prevalent chronic diseases worldwide (Selwitz et al., 2007). It is a result of complex interaction over time between acid-producing bacteria and fermentable carbohydrate, and other host factors including saliva (Selwitz et al., 2007). Penetration of oral bacteria into the dentin layer triggers inflammatory responses in the dental pulp (Farges et al., 2015), which responds to injury or inflammation according to the severity of infection (mild versus deep caries). The inflammatory process is characterized by inflammatory cell infiltrate and immature progenitor cell recruitment (Chen et al., 2006). DPC biology is known to be affected by caries, but further studies are needed to determine in detail the causative molecular mechanisms associated with these processes. DPCs themselves have immunomodulatory effects and have been successfully isolated from inflamed dental pulps (Alongi et al., 2010). Interestingly, these cells showed high expression levels of some mesenchymal stem cell markers (Chen and Thouas, 2011) although there is some controversy in relation to their differentiation potential in comparison with cells isolated from normal pulp (McLachlan et al., 2004; Ma et al., 2012). cDPCs were found to have a higher mineralization potential as well as a higher expression of gene associated with osteogenesis and/or odontogenesis, compared to hDPCs (Alkharobi et al., 2017). The rate of dentin repair/regeneration is closely related to the population size of remaining vital odontoblasts or newly differentiated odontoblast-like cells. If the inflammation is not too severe and/or is rapidly controlled, then innate pulp repair mechanisms can generally suffice for regeneration of the tissue (Farges et al., 2015). Carious teeth are usually extracted and discarded; however, as indicated above these cells are also a potential source of DPCs (Alongi et al., 2010; Werle et al., 2016).

The insulin-like growth factor (IGF) axis comprises two polypeptide growth factors (IGF-1 and IGF-2), two cell surface receptors (IGF-1R and IGF-2R), and six high affinity, soluble IGF binding proteins (IGFBP1–6) The IGF axis is known to play a role in the differentiation of progenitor cells into dental mineralized tissue (Gotz et al., 2006a; Chen et al., 2010; Abreu et al., 2013) being associated with the induction of enamel bio-mineralization (Takahashi et al., 1998), the differentiation of dental pulp cells (DPCs) (Onishi et al., 1999), and reparative dentinogenesis (Lovschall et al., 2001). Liposomal delivery of IGF1 along with calcium hydroxide into the tooth socket enhanced the deposition of osteodentin-like matrix around dental implants (Tziafas et al., 1998). Similarly, IGF-1 in combination with platelet-derived growth factor and calcium hydroxide improved healing of apical tooth perforations in a canine model. Further evidence suggested that IGF-1 regulated the balance between odontogenesis and osteogenesis in apical papilla stem cells (Wang et al., 2012) and IGF-1 enhances odontogenic differentiation and deposition of extracellular matrix in differentiating DPCs (Li et al., 1998; Onishi et al., 1999). IGF-2 was reported to be expressed by DPCs at both gene and protein levels, although its function in this tissue is still largely unknown (Shi et al., 2001). IGF-1R showed higher expression in teeth with incomplete root development, suggesting a role for IGF-1 in this process (Caviedes-Bucheli et al., 2004). IGFBP-1, -3, -5, and -6 have also been detected in DPCs isolated from healthy premolars and third molars (Gotz et al., 2006b).

In our previous study, we analyzed IGF axis gene expression in DPCs and demonstrated a novel role of IGFBP-2 and -3 in coordinating mineralized matrix formation (Alkharobi et al., 2016). A more recent study from our laboratory partially characterized DPCs from teeth with superficial caries (cDPCs) and showed that their potential to differentiate to a matrix mineralizing phenotype is enhanced by exposure to the mild inflammation characteristic of superficial caries (Alkharobi et al., 2017). In the current study, we aimed to determine whether the changes in IGF axis expression characteristic of differentiating hDPCs were also seen during differentiation of cDPCs.

## Materials and Methods

### Tissue Culture

Freshly extracted carious fully erupted third molars were collected from three adult patients (20–40 years of age) at the outpatient’s dental clinic of Leeds Dental Institute. Teeth were obtained through Leeds Dental and Skeletal tissue bank (under the approval of the Leeds University Dental Research Ethics Committee number 130111/AH/75), with patients’ informed consent (Alkharobi et al., 2017). Carious lesions in this study were chosen based on the depth of the decay in the dentin layer which was assessed in this group during the sectioning of the teeth. Teeth with more than 2 mm of sound dentin measured from the edge of carious lesion to the pulp tissue, were included in this study and categorized as shallow caries lesions (McLachlan et al., 2004; Gallego et al., 2010). This was assessed visually and using a WHO periodontal probe. Single cell suspensions were created by collagenase digestion of the isolated pulps as previously described (Alkharobi et al., 2016, 2017). cDPCs at passage 4 were cultured in 6-well plates at 10^5^ cells/well under basal conditions [α-MEM supplemented with 20% (v/v) FBS, 200 mM l-glutamine, and 100 unit/ml Pen Strep]. When the cells reached 80% confluence, they were cultured in triplicate under basal or mineralizing/osteogenic conditions (basal medium + 10 nM dexamethasone and 100 µM of l-ascorbic acid).

### qRT-PCR

RNeasy^®^ Mini Kit (Qiagen, UK) was used to extract and purify mRNA exactly according to the manufacturer’s protocol. mRNA quality was confirmed by monitoring A260/280 ratios and 1 µg mRNA was used for first strand cDNA synthesis using High Capacity mRNA to cDNA kit (Applied Biosystems, UK) exactly according to the manufacturer’s protocol. qRT-PCR reactions were conducted in a total volume of 20 µl using TaqMan probes and primers. Reactions were amplified using the Roche 480 Light Cycler^®^. Gene expression analysis was carried out using the ΔΔCt method, which is plotted as ordinate and indicates fold differences in expression mineralizing versus basal. TaqMan assay identifiers for each gene are presented in Table 1.

**Table 1:** Assay identifiers for TaqMan qRT-PCR.

Gene name	TaqMan^®^Gene expression assay identifier
GAPDH	Hs99999905_m1
IGF1	Hs01547656_m1
IGF2	Hs04188276_m1
IGF1R	Hs00609566_m1
IGF2 R	Hs00974474_m1
IGFBP1	Hs00236877_m1
IGFBP2	Hs01040719_m1
IGFBP3	Hs00426289_m1
IGFBP4	Hs01057900_m1
IGFBP5	Hs00181213_m1
IGFBP6	Hs00181853_m1

*Further details available at www.appliedbiosystems.com*.

### ELISA

IGF binding protein-2 and IGFBP-3 concentrations in conditioned media were determined by enzyme-linked immunosorbent assay (R&D Systems, UK) exactly according to the manufacturer’s protocol and as described previously (Alkharobi et al., 2016). 1 ml of conditioned medium (24 h conditioning period; 3 × 10^5^ cells) was collected from basal and mineralizing cultures at both 1- and 3-week time points, centrifuged briefly to remove cell debris and stored at −80°C prior to assay. Unconditioned medium was used as a negative control. Standard curves for ELISA were constructed in unconditioned medium and were linear between 0.0625 and 4 ng/ml (IGFBP-2) and 0.781–50 ng/ml (IGFBP-3). Samples of conditioned medium were appropriately diluted in unconditioned medium (total volume = 50 μl) to fall in this region of the standard curve. Assays were performed on media conditioned by cDPC cultures derived from three separate donors and were typically assayed as technical triplicates. Data are presented as ng/ml (mean ± SD).

### Statistics

Statistical analysis was carried out for gene expression results on individual donors (*n* = 3) and for global gene expression data, using one way ANOVA followed by Bonferroni multiple comparison tests, using Graph Pad Prism software (v 6). Differences were considered significant when *p* values were < 0.05.

Statistical analysis of IGFBP-2 and IGFBP-3 protein concentrations in basal and osteogenic conditioned medium was by unpaired *t*-test and was considered significant at *p* < 0.05 (B versus O). Graphpad Prism version 6.

## Results

### IGF Axis Expression

**Figure 1:**
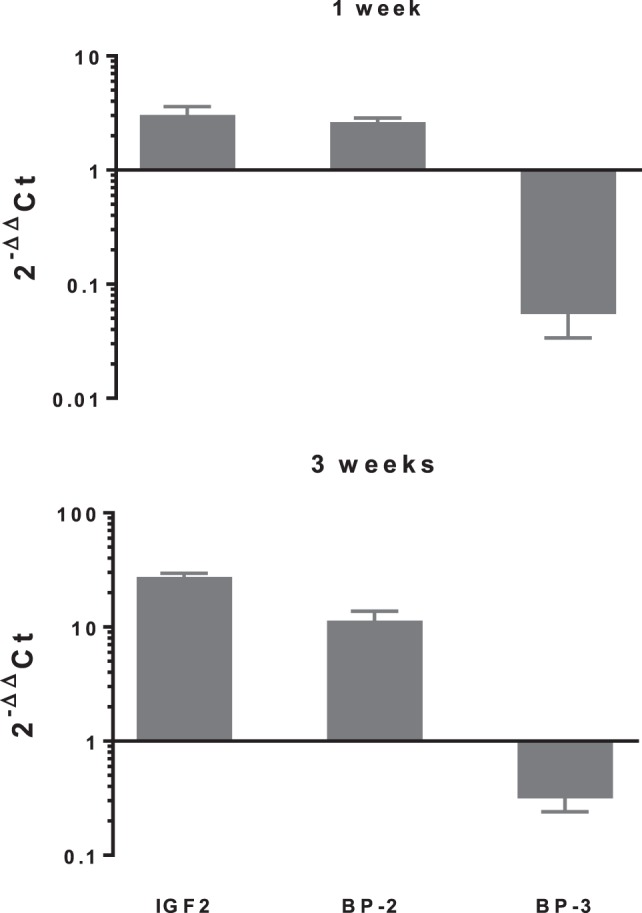
Changes in expression of selected insulin-like growth factor (IGF) axis genes in cDPCs cultured under osteogenic conditions. Relative changes in selected IGF axis gene expression in cDPC cultured in monolayers under osteogenic conditions for 1 and 3 weeks are indicated. Cultures were derived from three donors and triplicate technical replicates were performed for each donor. Data are presented as mean ± SD (*n* = 3). The relative gene expression was normalized to corresponding controls cultured under basal conditions and is expressed as 2^−ΔΔCt^.

**Figure 2:**
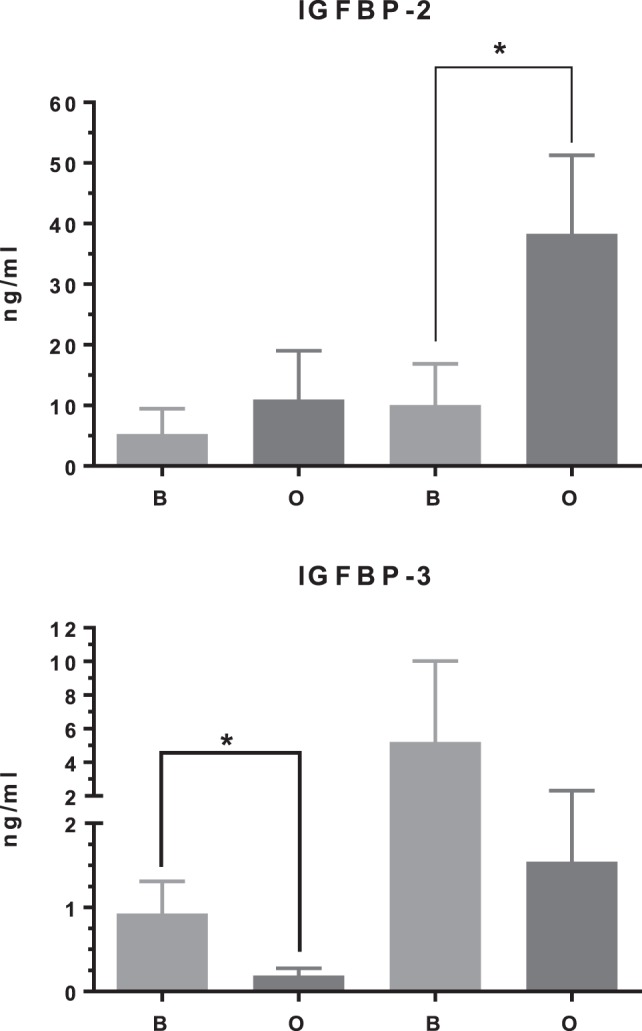
IGF binding protein (IGFBP)-2 and IGFBP-3 protein concentration in cDPC cultured under basal or mineralizing conditions. Media was conditioned by cells from three separate donors grown under basal or osteogenic conditions at both 1- and 3-week time points. Assays were performed as technical triplicates for each donor and are presented as mean ± SEM (*n* = 7–9) in nanogram per milliliter for each protein(*p<0.01).

In differentiating cDPCs only *IGF2* and *IGFBP-2* were upregulated consistently at week 1 and week 3 in all donors (Figure 1). At the week 1 time point, both *IGF2* and *IGFBP-2* were upregulated approximately threefold. At the 3 week time point, IGF2 expression was upregulated almost 30-fold and *IGFBP-2* approximately 10-fold. Conversely, *IGBP-3* was consistently downregulated in cDPCs from all donors (Figure 1). At the 1 week time point, *IGFBP-3* was downregulated 20-fold at the 3-week time point around 4-fold. Changes in *IGFBP-2* and *IGFBP-3* mRNA expression under osteogenic conditions in cDPCs were reflected in corresponding changes in protein concentration in conditioned medium (Figure 2). For IGFBP-2 at week 1 protein concentrations in conditioned medium were 5.3 ± 1.5 versus 10.1 ± 2.4 ng/ml B versus O (*n* = 7–9 mean ± SEM). At the 3 week time point, IGFBP-2 concentrations were 11.0 ± 2.7 versus 38.3 ± 4.9 ng/ml B versus O (*n* = 7–8 mean ± SEM). At the week 3 time point, this difference was considered significant *p* < 0.05 and at the week 1 time point approached significance (*p* = 0.11). For IGFBP-3 protein concentrations in conditioned medium at week 1 time point were 0.93 ± 0.13 versus 0.10 ± 0.03 ng/ml B versus O (*n* = 9 mean ± SEM). At the 3 week time point, protein concentrations were 5.2 ± 1.7 versus 1.5 ± 0.27 ng/ml B versus O (*n* = 8 ± SEM). At 1 week, this difference was significant at *p* < 0.05 and at week 3 was close to significance (*p* = 0.051). For IGF1R, IGF2R, IGFBP-4, -5, and -6 there was no significant difference in mRNA or protein concentrations basal versus osteogenic (data not shown). In our hands, expression of both IGF1 and IGFBP-1 was very low (Ct > 35) under both basal and osteogenic conditions (data not shown).

## Discussion

The expression of the IGF axis under mineralizing/osteogenic conditions in cDPCs has not previously been reported. The current study showed that IGF-1 was expressed at very low levels by cDPCs confirming our previous observations in hDPCs. Literature concerning IGF1 expression in dental tissue derived cells is scarce and at times contradictory. Joseph et al. showed *IGF1* mRNA expression in cells derived from the apical loop and in secretory ameloblasts in the continuously erupting rat incisor model. However, in agreement with our data, *IGF1* mRNA was low or absent in dental pulp derived cells (Joseph et al., 1996). However, using immunohistochemical techniques, IGF1 peptide was identified in human dental pulps with expression levels correlated with the stage of root development (Caviedes-Bucheli et al., 2009). It is well established that DPC in culture respond to exogenous IGF1 (Li et al., 1998; Onishi et al., 1999), therefore, further investigation of endogenous IGF1 expression in DPCs would allow some conclusion regarding autocrine/paracrine versus endocrine actions of IGF1 in dental pulp tissue. *IGF-2* mRNA expression demonstrated consistent upregulation in cDPCs under mineralizing/osteogenic conditions at 1 and 3 week time points again confirming previous observations in hDPCs. We have recently confirmed that IGF2 peptide is present at 10-fold higher concentrations in differentiated DPCs compared to cells grown under basal conditions (Al-Khafaji et al. submitted for publication). We believe our data represent the first demonstration of increased *IGF2* expression under mineralization conditions in DPCs and confirm previous findings that IGF2 is the main IGF species expressed in dental pulp (Reichenmiller et al., 2004). IGF2 may be sequestered in the matrix during dentin formation and released during demineralization of dentin. This may to some extent explain the pattern of *IGF-2* gene expression in cDPCs observed in the current study. Interestingly, *IGF-2* was highly expressed in DPCs isolated from deciduous teeth, where the pulp was exposed during the removal of proximal caries.

We have demonstrated reproducible upregulation of *IGFBP-2* gene expression in cDPCs under mineralizing/osteogenic conditions at 1 and 3 week time points. These findings were mirrored in the results for protein concentrations in conditioned medium. As such these findings confirm our previous observations in hDPCs (Alkharobi et al., 2016). There is very little data on the expression and activity of any of the *IGFBP* gene family in dental tissues, however, the involvement of *IGFBP-2* in enhanced osteogenesis was suggested by the observation of skeletal thickening associated with elevated serum IGFBP-2 levels in hepatitis-associated osteosclerosis (Khosla et al., 1998). Although there are some reports of inhibitory effects of IGFBP-2 on bone size, mass, and proliferation of rat calvarias derived cells (Feyen et al., 1991; Eckstein et al., 2002), consensus opinion appears to favor an anabolic role of IGFBP-2 in osteogenesis either complexed with IGFs or acting independently of the growth factor (Conover and Khosla, 2003; Hamidouche et al., 2010).

In this study, *IGFBP-3* was consistently downregulated in cDPCs under mineralizing conditions again confirming previous data in hDPCs. Early studies suggested that the expression of *IGFBP-3* can be regulated by glucocorticoids such as dexamethasone. Glucocorticoids inhibit *IGFBP-3* expression in hepatocytes (Villafuerte et al., 1995) and fibroblasts (Conover et al., 1995), although this synthetic glucocorticoid is also reported to upregulate *IGFBP-3* expression *in vivo*. Based on these reports, the levels of *IGFBP-3* in the current study might be downregulated due to the effect of dexamethasone in the mineralizing differentiation culture medium. Another group who investigated *IGFBP-3* expression in an odontoblast-like cell line cultured in non-dexamethasone containing medium confirmed the expression of *IGFBP-3* at later stages (25 days) of differentiation (Caton et al., 2007). However, Jia and Heersche (2002) reported that *IGFBP-3* gene expression was lower in dexamethasone-treated cultures at day 20 but higher at day 8 and day 14 than in basal cultures. The use of dexamethasone free differentiation medium may, therefore, be indicated for future experiments and it would be interesting to compare the results of such experiments with those reported in the current study.

In contrast to our findings, Reichenmiller et al. (2004) reported that IGFBP-2 concentrations decreased slightly, while IGFBP-3 increased during differentiation of human pulp cells isolated from healthy third molars. There are several possible explanations for this contradiction, Reichenmiller et al. (2004) assayed the cell extracts (and thus intracellular IGFBPs) to determine the protein concentration of IGFBP-2 and IGFBP-3, while in the current study these proteins were measured in conditioned media. IGFBPs are essentially secreted proteins, and therefore media conditioned by DPCs are considered more appropriate sources to investigate IGFBP-2 and IGFBP-3 concentrations *in vitro*. In addition, Reichenmiller et al. (2004) did not treat the cells with dexamethasone, or ascorbic acid but simply allowed confluent cells to differentiate in basal medium. Furthermore, our current findings showing IGFBP-2 upregulation in mineralizing medium containing dexamethasone confirms previous observations (Jia and Heersche, 2002). In the current study, IGFBP-2 and IGFBP-3 levels were higher in cDPCs compared to cells isolated form healthy teeth (Alkharobi et al., 2016). The mild inflammatory environment associated with superficial caries is known to activate NF-kB (Lawrence, 2009) and following nuclear translocation NF-kB activates expression of numerous target genes including IGFBPs (Shoelson et al., 2006).

## Conclusion

This study confirms that the changes in IGF expression previously reported in DPCs derived from healthy dental pulps are replicated in DPCs derived from carious lesions and suggests that similar IGF axis-based regulation of mineralization may occur in cDPCs.

## Ethics Statement

In this study we used freshly extracted carious fully erupted third molars collected from adult patients (20–40 years of age) at the outpatient’s dental clinic of Leeds Dental Institute. Teeth were obtained through Leeds Dental and Skeletal tissue bank (LDI Research Tissue Bank; 130111/AH/75), with patients’ informed consent.

## Author Contributions

HA and HA-K carried out the experimental work and the analysis of the data. JB and RE-G conceived the study contributed to the experimental design and analysis of data. JB, RE-G, and DD supervised the project. RE-G wrote the manuscript.

## Conflict of Interest Statement

The authors declare that the research was conducted in the absence of any commercial or financial relationships that could be construed as a potential conflict of interest.

## References

[B1] Abreu F. A. Ferreira C. L. Silva G. A. Paulo Cde O. Miziara M. N. Silveira F. F. (2013). Effect of PDGF-BB, IGF-I growth factors and their combination carried by liposomes in tooth socket healing. Braz. Dent. J. 24, 299–307. 10.1590/0103-6440201302238 24173245

[B2] Alkharobi H. Alhodhodi A. Hawsawi Y. Alkafaji H. Devine D. El-Gendy R. (2016). IGFBP-2 and -3 co-ordinately regulate IGF1 induced matrix mineralisation of differentiating human dental pulp cells. Stem Cell Res. 17, 517–522. 10.1016/j.scr.2016.09.026 27776273 PMC5153425

[B3] Alkharobi H. Beattie J. Meade J. Devine D. El-Gendy R. (2017). Dental pulp cells isolated from teeth with superficial caries retain an inflammatory phenotype and display an enhanced matrix mineralization potential. Front. Physiol. 8:244. 10.3389/fphys.2017.00244 28503150 PMC5408163

[B4] Alongi D. J. Yamaza T. Song Y. Fouad A. F. Romberg E. E. Shi S. (2010). Stem/progenitor cells from inflamed human dental pulp retain tissue regeneration potential. Regen. Med. 5, 617–631. 10.2217/rme.10.30 20465527 PMC3035701

[B5] Caton J. Bringas P. Jr. Zeichner-David M. (2007). Establishment and characterization of an immortomouse-derived odontoblast-like cell line to evaluate the effect of insulin-like growth factors on odontoblast differentiation. J. Cell. Biochem. 100, 450–463. 10.1002/jcb.21053 16927272

[B6] Caviedes-Bucheli J. Canales-Sanchez P. Castrillon-Sarria N. Jovel-Garcia J. Alvarez-Vasquez J. Rivero C. (2009). Expression of insulin-like growth factor-1 and proliferating cell nuclear antigen in human pulp cells of teeth with complete and incomplete root development. Int. Endod. J. 42, 686–693. 10.1111/j.1365-2591.2009.01568.x 19467045

[B7] Caviedes-Bucheli J. Munoz H. R. Rodriguez C. E. Lorenzana T. C. Moreno G. C. Lombana N. (2004). Expression of insulin-like growth factor-1 receptor in human pulp tissue. J. Endod. 30, 767–769. 10.1097/01.DON.0000134203.65706.8F 15505506

[B8] Chen L. Jiang W. Huang J. He B. C. Zuo G. W. Zhang W. (2010). Insulin-like growth factor 2 (IGF-2) potentiates BMP-9-induced osteogenic differentiation and bone formation. J. Bone Miner. Res. 25, 2447–2459. 10.1002/jbmr.133 20499340 PMC3179288

[B9] Chen Q. Z. Thouas G. A. (2011). Fabrication and characterization of sol-gel derived 45S5 Bioglass (R)-ceramic scaffolds. Acta Biomater. 7, 3616–3626. 10.1016/j.actbio.2011.06.005 21689791

[B10] Chen S. C. Marino V. Gronthos S. Bartold P. M. (2006). Location of putative stem cells in human periodontal ligament. J. Periodont. Res. 41, 547–553. 10.1111/j.1600-0765.2006.00904.x 17076780

[B11] Conover C. A. Durham S. K. Zapf J. Masiarz F. R. Kiefer M. C. (1995). Cleavage analysis of insulin-like growth factor (IGF)-dependent IGF-binding protein-4 proteolysis and expression of protease-resistant IGF-binding protein-4 mutants. J. Biol. Chem. 270, 4395–4400. 10.1074/jbc.270.9.4395 7533161

[B12] Conover C. A. Khosla S. (2003). Role of extracellular matrix in insulin-like growth factor (IGF) binding protein-2 regulation of IGF-II action in normal human osteoblasts. Growth Horm. IGF Res. 13, 328–335. 10.1016/S1096-6374(03)00092-3 14624766

[B13] Eckstein F. Pavicic T. Nedbal S. Schmidt C. Wehr U. Rambeck W. (2002). Insulin-like growth factor-binding protein-2 (IGFBP-2) overexpression negatively regulates bone size and mass, but not density, in the absence and presence of growth hormone/IGF-I excess in transgenic mice. Anat. Embryol. (Berl) 206, 139–148. 10.1007/s00429-002-0282-5 12478375

[B14] Farges J. C. Alliot-Licht B. Renard E. Ducret M. Gaudin A. Smith A. J. (2015). Dental pulp defence and repair mechanisms in dental caries. Mediators Inflamm. 2015, 230251. 10.1155/2015/230251 26538821 PMC4619960

[B15] Feyen J. H. Evans D. B. Binkert C. Heinrich G. F. Geisse S. Kocher H. P. (1991). Recombinant human [Cys281]insulin-like growth factor-binding protein 2 inhibits both basal and insulin-like growth factor I-stimulated proliferation and collagen synthesis in fetal rat calvariae. J. Biol. Chem. 266, 19469–19474. 1717466

[B16] Gallego L. Junquera L. Meana A. Garcia E. Garcia V. (2010). Three-dimensional culture of mandibular human osteoblasts on a novel albumin scaffold: growth, proliferation, and differentiation potential in vitro. Int. J. Oral Maxillofac. Implants 25, 699–705. 20657864

[B17] Gotz W. Kunert D. Zhang D. Kawarizadeh A. Lossdorfer S. Jager A. (2006a). Insulin-like growth factor system components in the periodontium during tooth root resorption and early repair processes in the rat. Eur. J. Oral Sci. 114, 318–327. 10.1111/j.1600-0722.2006.00381.x 16911103

[B18] Gotz W. Heinen M. Lossdorfer S. Jager A. (2006b). Immunohistochemical localization of components of the insulin-like growth factor system in human permanent teeth. Arch. Oral Biol. 51, 387–395. 10.1016/j.archoralbio.2005.10.005 16321360

[B19] Hamidouche Z. Fromigue O. Nuber U. Vaudin P. Pages J. C. Ebert R. (2010). Autocrine fibroblast growth factor 18 mediates dexamethasone-induced osteogenic differentiation of murine mesenchymal stem cells. J. Cell. Physiol. 224, 509–515. 10.1002/jcp.22152 20432451

[B20] Jia D. Heersche J. N. (2002). Expression of insulin-like growth factor system constituents in differentiating rat osteoblastic cell populations. Growth Horm. IGF Res. 12, 399–410. 10.1016/S1096-6374(02)00117-X 12423625

[B21] Joseph B. K. Savage N. W. Daley T. J. Young W. G. (1996). In situ hybridization evidence for a paracrine/autocrine role for insulin-like growth factor-I in tooth development. Growth Factors 13, 11–17. 10.3109/08977199609034563 8962715

[B22] Khosla S. Hassoun A. A. Baker B. K. Liu F. Zein N. N. Whyte M. P. (1998). Insulin-like growth factor system abnormalities in hepatitis C-associated osteosclerosis. Potential insights into increasing bone mass in adults. J. Clin. Invest. 101, 2165–2173. 10.1172/JCI1111 9593772 PMC508804

[B23] Lawrence T. (2009). The nuclear factor NF-kappaB pathway in inflammation. Cold Spring Harb. Perspect. Biol. 1, a001651 10.1101/cshperspect.a001651 20457564 PMC2882124

[B24] Li H. Bartold P. M. Zhang C. Z. Clarkson R. W. Young W. G. Waters M. J. (1998). Growth hormone and insulin-like growth factor I induce bone morphogenetic proteins 2 and 4: a mediator role in bone and tooth formation? Endocrinology 139, 3855–3862. 10.1210/endo.139.9.6211 9724040

[B25] Lovschall H. Fejerskov O. Flyvbjerg A. (2001). Pulp-capping with recombinant human insulin-like growth factor I (rhIGF-I) in rat molars. Adv. Dent. Res. 15, 108–112. 10.1177/08959374010150010301 12640754

[B26] Ma D. Gao J. Yue J. Yan W. Fang F. Wu B. (2012). Changes in proliferation and osteogenic differentiation of stem cells from deep caries in vitro. J. Endod. 38, 796–802. 10.1016/j.joen.2012.02.014 22595115

[B27] McLachlan J. L. Sloan A. J. Smith A. J. Landini G. Cooper P. R. (2004). S100 and cytokine expression in caries. Infect. Immun. 72, 4102–4108. 10.1128/IAI.72.7.4102-4108.2004 15213155 PMC427449

[B28] Onishi T. Kinoshita S. Shintani S. Sobue S. Ooshima T. (1999). Stimulation of proliferation and differentiation of dog dental pulp cells in serum-free culture medium by insulin-like growth factor. Arch. Oral Biol. 44, 361–371. 10.1016/S0003-9969(99)00007-2 10348363

[B29] Reichenmiller K. M. Mattern C. Ranke M. B. Elmlinger M. W. (2004). IGFs, IGFBPs, IGF-binding sites and biochemical markers of bone metabolism during differentiation in human pulp fibroblasts. Horm. Res. 62, 33–39. 10.1159/000078747 15166484

[B30] Selwitz R. H. Ismail A. I. Pitts N. B. (2007). Dental caries. Lancet 369, 51–59. 10.1016/S0140-6736(07)60031-2 17208642

[B31] Shi S. Robey P. G. Gronthos S. (2001). Comparison of human dental pulp and bone marrow stromal stem cells by cDNA microarray analysis. Bone 29, 532–539. 10.1016/S8756-3282(01)00612-3 11728923

[B32] Shoelson S. E. Lee J. Goldfine A. B. (2006). Inflammation and insulin resistance. J. Clin. Invest. 116, 1793–1801. 10.1172/JCI29069E1 16823477 PMC1483173

[B33] Takahashi K. Yamane A. Bringas P. Caton J. Slavkin H. C. Zeichner-David M. (1998). Induction of amelogenin and ameloblastin by insulin and insulin-like growth factors (IGF-I and IGF-II) during embryonic mouse tooth development in vitro. Connect. Tissue Res. 38, 269–78; discussion 295–303. 10.3109/03008209809017047 11063034

[B34] Tziafas D. Alvanou A. Papadimitriou S. Gasic J. Komnenou A. (1998). Effects of recombinant basic fibroblast growth factor, insulin-like growth factor-II and transforming growth factor-beta 1 on dog dental pulp cells in vivo. Arch. Oral Biol. 43, 431–444. 10.1016/S0003-9969(98)00026-0 9717581

[B35] Villafuerte B. C. Koop B. L. Pao C. I. Phillips L. S. (1995). Glucocorticoid regulation of insulin-like growth factor-binding protein-3. Endocrinology 136, 1928–1933. 10.1210/endo.136.5.7536659 7536659

[B36] Wang S. Mu J. Fan Z. Yu Y. Yan M. Lei G. (2012). Insulin-like growth factor 1 can promote the osteogenic differentiation and osteogenesis of stem cells from apical papilla. Stem Cell Res. 8, 346–356. 10.1016/j.scr.2011.12.005 22286010

[B37] Werle S. B. Lindemann D. Steffens D. Demarco F. F. de Araujo F. B. Pranke P. (2016). Carious deciduous teeth are a potential source for dental pulp stem cells. Clin. Oral Investig. 20, 75–81. 10.1007/s00784-015-1477-5 25898896

